# High-Throughput Direct Writing of Metallic Micro- and Nano-Structures by Focused Ga^+^ Beam Irradiation of Palladium Acetate Films

**DOI:** 10.1021/acsami.2c05218

**Published:** 2022-06-07

**Authors:** Alba Salvador-Porroche, Lucía Herrer, Soraya Sangiao, Patrick Philipp, Pilar Cea, José María De Teresa

**Affiliations:** † Instituto de Nanociencia y Materiales de Aragón (INMA), CSIC-Universidad de Zaragoza, Zaragoza 50009, Spain; ‡ Laboratorio de Microscopías Avanzadas (LMA), Universidad de Zaragoza, Zaragoza 50018, Spain; § Advanced Instrumentation for Nano-Analytics (AINA), MRT Department, Luxembourg Institute of Science and Technology (LIST), 41 rue du Brill, Belvaux 4422, Luxembourg

**Keywords:** focused ion beams, spin-coated organometallic films, electrical contacts, nanogap electrodes, large-area meshes

## Abstract

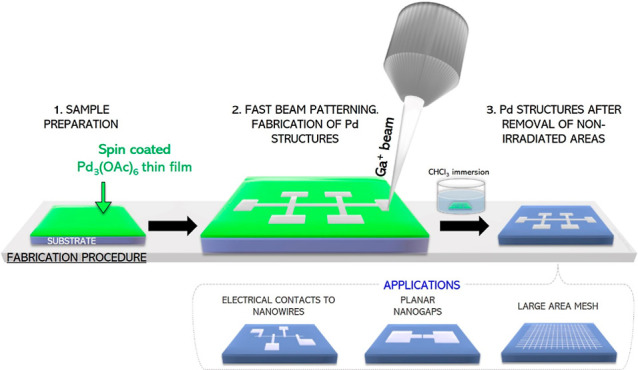

Metallic nanopatterns are ubiquitous in applications that exploit the electrical conduction at the nanoscale, including interconnects, electrical nanocontacts, and small gaps between metallic pads. These metallic nanopatterns can be designed to show additional physical properties (optical transparency, plasmonic effects, ferromagnetism, superconductivity, heat evacuation, etc.). For these reasons, an intense search for novel lithography methods using uncomplicated processes represents a key on-going issue in the achievement of metallic nanopatterns with high resolution and high throughput. In this contribution, we introduce a simple methodology for the efficient decomposition of Pd_3_(OAc)_6_ spin-coated thin films by means of a focused Ga^+^ beam, which results in metallic-enriched Pd nanostructures. Remarkably, the usage of a charge dose as low as 30 μC/cm^2^ is sufficient to fabricate structures with a metallic Pd content above 50% (at.) exhibiting low electrical resistivity (70 μΩ·cm). Binary-collision-approximation simulations provide theoretical support to this experimental finding. Such notable behavior is used to provide three proof-of-concept applications: (i) creation of electrical contacts to nanowires, (ii) fabrication of small (40 nm) gaps between large metallic contact pads, and (iii) fabrication of large-area metallic meshes. The impact across several fields of the direct decomposition of spin-coated organometallic films by focused ion beams is discussed.

## Introduction

Metallic nanopatterns are important building blocks in modern technology with fundamental relevance in the construction of interconnects for microelectronics,^1^ conductive nanomeshes for organic photovoltaics^2^ and for transparent conductive electrodes,^3^ organic light-emitting diodes for flexible electronics,^4^ plasmonic nanostructures for sensing^5^ and for lithography,^6^ nanogap electrodes for optical applications^7^ and for molecular electronics,^8^ electrical contacts to nanowires for sensing^9^ and for quantum technologies,^10^ and so forth.

The existing fabrication methods of these metallic nanopatterns are based either on chemical or physical approaches or on hybrid physical/chemical methodologies, with each technique leading to different features in terms of resolution, throughput, cost, and so forth.^11,12^ Whereas bottom-up chemical methods (such as those relying on self-assembly and self-organization) generally excel in throughput and cost,^13,14^ top-down lithography techniques based on physical techniques (such as optical/electron/ion beam lithography in combination with thin-film techniques) allow for the control of the dimensions of the nanopattern with great accuracy.^15,16^ Importantly, hybrid approaches (combining chemical and physical techniques) can benefit from the virtues of both, with two examples being nanosphere lithography and directed block-copolymer lithography.^17,18^ Without pretending to be comprehensive, a few paradigmatic examples of advanced nanofabrication methods to create metallic nanopatterns can be discussed. For instance, mature multi-step techniques based on resists, such as optical lithography,^19^ electron-beam lithography (EBL),^20^ and nanoimprint lithography,^21^ are capable of patterning metals with a high resolution and throughput. Direct patterning of metals using a focused ion beam (FIB)^22^ or by scanning probe lithography^23^ has demonstrated high-resolution capabilities, albeit at the expense of low throughput. Moreover, less conventional approaches have been used in specific applications, such as the fabrication of ultra-small gaps through sketch-and-peel lithography combined with transfer printing^24^ and three-dimensional metallic nano-architectures by means of two-photon lithography^25^ or focused electron beam-induced deposition.^26^

Without undermining the power of such advanced methods to fabricate metallic nanopatterns, it is worth exploring other nanofabrication methods that could combine virtues such as simplicity, high resolution, and high throughput. In this context, the direct decomposition of organometallic films by focused charged beams is an interesting route to explore. The advantage of this technique lies in the fact that after spin coating of the organometallic film and the subsequent electron or ion irradiation, the metallic (Pd, Ag, Au, Ir, etc.) nanopattern is readily revealed by dissolving non-irradiated areas in a cleaning solvent.^27−31^ Thus, no sacrificial resist layer is necessary and, in the end, the organometallic film precursor becomes the functional material. However, in all cases reported in the literature so far, a post-processing annealing step is required to obtain low electrical resistivity of the nanopatterned material.^31−35^

**Figure 1:**
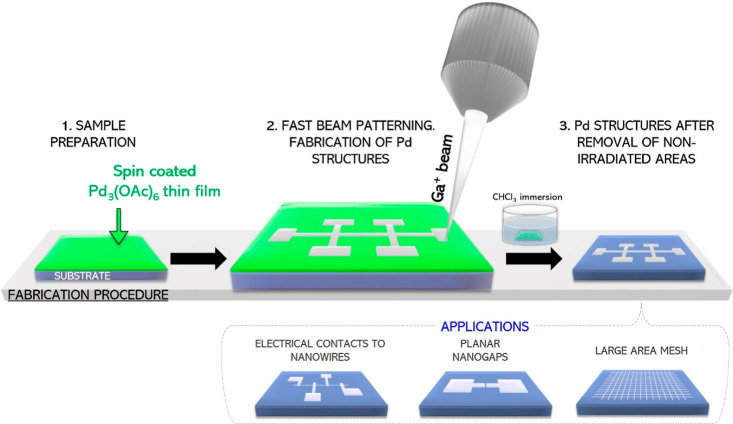
Sketch of the fabrication process: (1) deposition of the Pd_3_(OAc)_6_ film by spin coating. (2) Formation of Pd nanostructures by Ga^+^ FIB. (3) Pd structures’ unveiling process with the removal of non-irradiated film areas. The three applications studied in this contribution are also sketched at the bottom of the figure.

Recently, our group has shown that by irradiating a Pd_3_(OAc)_6_ thin film with a high electron dose, it is possible to produce metallic nanostructures without the need of post-processing annealing steps.^36^ Unfortunately, the high dose required (30,000 μC/cm^2^) limits the applications of this approach to small-area patterning or low-throughput device fabrication. Motivated by the search for a high-throughput and annealing-free patterning process of Pd_3_(OAc)_6_ thin films, we have investigated whether Ga^+^ FIB irradiation could be effective toward a decrease in the required charge dose without putting in jeopardy the low electrical resistivity of the nanostructure. An illustration of the work here presented is summarized in Figure 1.

Remarkably, we have found that only 30 μC/cm^2^ Ga^+^ irradiation is required to directly obtain metallic structures with a resistivity of 70 μΩ·cm. Importantly, this finding represents a substantial improvement with respect to all previous existing results and opens the route to large-area and high-throughput device applications. In order to put this result in context, it is worth mentioning that standard EBL processes make use of PMMA resists that need irradiation doses up to a few hundred μC/cm^2^,^37^ and FIB-induced deposition of metals (Pt, W, and Co) requires irradiation doses in the range of 10,000 μC/cm^2^.^38^

For clarification purposes, this article is organized as follows. First, the optimization of the ion irradiation procedure employed to obtain functional Pd nanostructures with low electrical resistivity will be presented. Second, simulations of the Ga^+^ irradiation process that decomposes the palladium acetate film will be provided. In the last part of the article, three applications of this nanolithography technique will be shown: (a) fabrication of electrical contacts to nanowires; (b) fabrication of small gaps between metal contacts, which in turn will serve to test the resolution of the technique; and (c) fabrication of conductive large-area meshes.

### Experimental Details

Ga^+^ FIB irradiations were carried out in a Helios NanoLab 650 (FEI company) on palladium acetate films with thicknesses in the 100–350 nm range; the palladium acetate films were previously deposited by spin coating onto Si/SiO_2_ substrates^36^ (see the sketch in Figure 1). Spin-coated films with thicknesses in the range of 100 to 200 nm were prepared by spreading 10 μL of a 0.09 M Pd_3_(OAc)_6_ filtered solution in chloroform. Furthermore, those with thicknesses in the range of 200 to 350 nm were prepared by spreading 15 μL of 0.2 M filtered solution. The spin-coating process in both cases includes two sequential steps: (1) 10 s at 3000 rpm and (2) 40 s at 4000 rpm. If the spin-coated film is too thick, some lift off and compositional problems could appear due to the inefficient decomposition of the bottom part of the film. The optimized parameters were 30 kV of the ion beam voltage, an ion beam current of 1.1, 7.7, or 24 pA (depending on the irradiation area), 200 ns of dwell time, and 0% of overlap. The organometallic films were decomposed by applying Ga^+^ doses in the 2–100 μC/cm^2^ range. After the developing step in chloroform, the resulting Pd nanostructures were characterized, and the optimal dose was chosen in terms of final application, which requires good electrical conductivities. Micro/nano pinholes have been found over the nanostructures. These have been associated with two contributing effects: (1) possible small bubbles/holes forming during the spin-coating procedure due to CHCl_3_ evaporation and (2) to the path created by the volatile components while being sublimated due to the beam exposure. Atomic force microscopy (AFM Veeco-Bruker Multimode 8) images of 25 μm^2^ microstructures fabricated at 20 and 30 μC/cm^2^. These images have been analyzed by using the Nanoscope V.1.40 software, obtaining a root-mean-square roughness value in the 0.9–1.7 nm range for pinhole-free smaller areas (see Figure S1).

Current-versus-voltage (*I*-*V*) measurements were performed at room temperature inside a Helios NanoLab 650 Dual Beam instrument (FEI company) using electrical microprobes (Kleindiek Nanotechnik GmbH) placed inside the chamber and a 6221 DC current source/2182A nanovoltmeter (Keithley Instruments) connected to the microprobes via a chamber feedthrough.

High-angle annular dark-field (HAADF) imaging and energy-dispersive X-ray spectroscopy (EDS) measurements were carried out along the nanostructure thickness by transmission electron microscopy (TEM) in an analytical Titan low-base instrument (FEI Company). HAADF images were obtained at 300 keV, and the energy resolution of the EDS experiments was ∼125 eV using the scanning transmission electron microscopy (STEM) mode.

X-ray photoelectron spectroscopy (XPS) was performed on a Kratos Axis UltraDLD spectrophotometer with a monochromatic Al Kα X-ray source (1486.6 eV) and a pass energy of 20 eV. Data treatment and further analysis were performed using the CasaXPS v.2.3.15 software. Binding energies have been referenced to the C 1s peak at 284.9 eV. Peak fitting was conducted by using standard line shapes GL(30) and LA(1.9,7, 2) for the asymmetric signal associated with metallic palladium.

Simulations based on binary collision approximation (BCA) were carried out using the SDTrimSP code^39^ to model the evolution of the precursor layer under Ga^+^ ion irradiation. This means that at a given time, only the interaction between two atoms is taken into account and many-body effects are neglected. The interatomic interactions were calculated using the KrC potential, and the electronic stopping was described by the Oen-Robinson model. Charges on atoms are not explicitly considered. The Gauss-Mehler method with 16 pivots was used for integration. The surface binding energy was calculated using the equation , where *sbe* is the surface binding energy of the current target, and  is the atomic surface binding energy of the chemical species *i*. The surface binding energy was calculated for any combination of two species used in this work, for example, gallium, silicon, palladium, carbon, hydrogen, and oxygen. The atomic densities of the different species were taken to be identical to the bulk values. Hence, the density of the simulated precursor layer might be above the value in the experiments. Neglecting the diffusion in SDTrimSP^40,41^ led to largely overestimated concentrations for carbon, hydrogen, and oxygen (*cf.*Supporting Information). The Monte Carlo code does not include temperature; therefore the diffusion processes are implemented using Fick’s law, where diffusion is described as a displacement per ion impact and is calculated by taking the ion flux in ions × cm^–2^ × s^–1^ into account. Optimization of the diffusion coefficients resulted in values of 10^6^ Å^4^/ion for hydrogen and oxygen and 2 × 10^5^ Å^4^/ion for carbon. The default displacement energies were used, that is, 12 eV for gallium, 13 eV for silicon, 26 eV for palladium, 25 eV for carbon, and 0.5 eV for hydrogen and oxygen. The precursor layer had an initial thickness of 110 nm. Ga^+^ irradiation was carried out at normal incidence with an impact energy of 30 keV. The maximum dose was set to 160 μC/cm^2^. The generation of secondary electrons is not considered in this approach.

## Results and Discussion

### Electrical Characterization by the Four-Probe Technique

**Figure 2:**
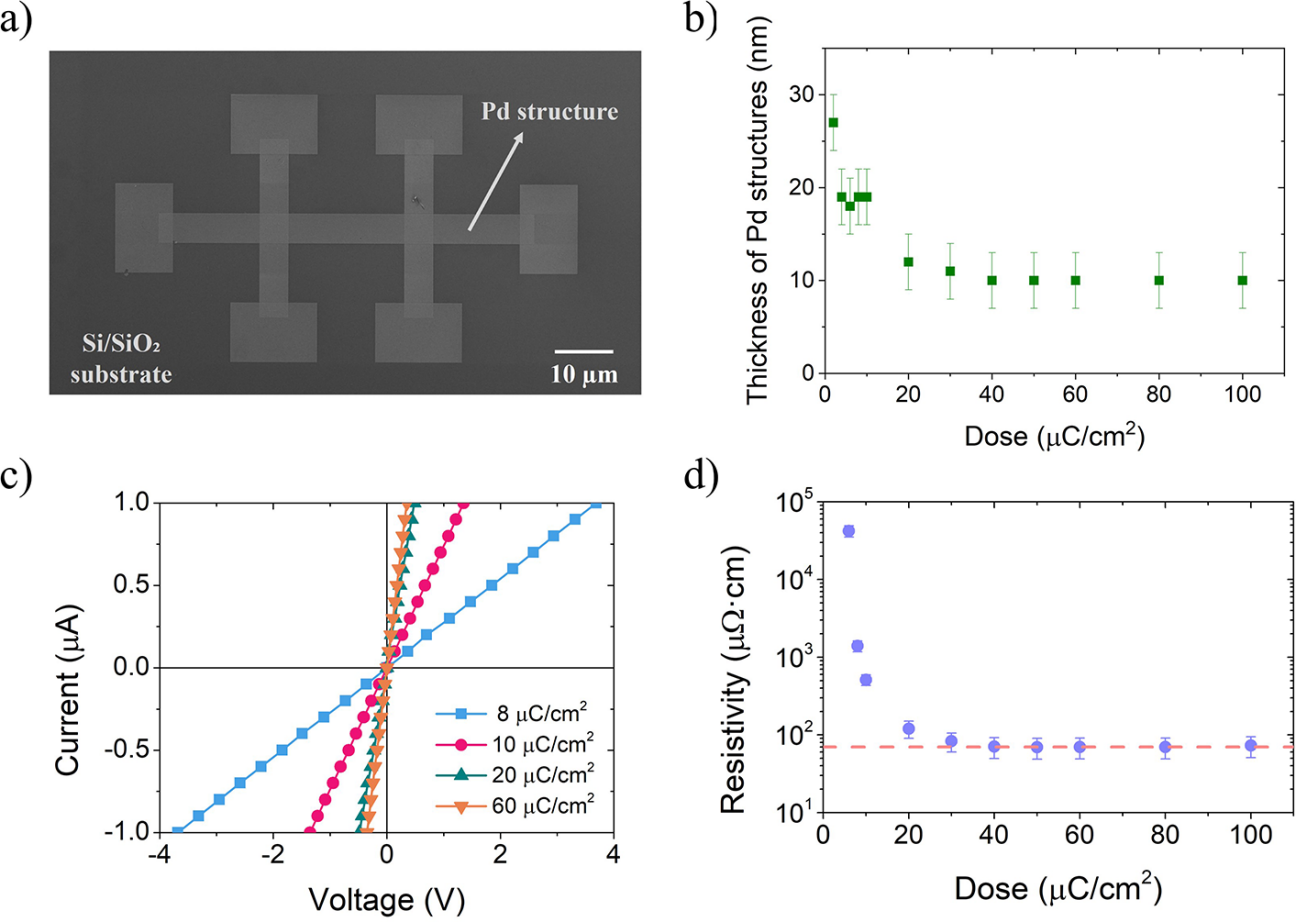
(a) SEM (scanning electron microscopy) micrograph of the measured Pd nanostructures. The total irradiated area, the beam current, and the irradiation time were ∼1500 μm^2^, 7.7 pA, and ∼20 s, respectively, which corresponds to an ion dose of 10 μC/cm^2^. (b) Thickness measurements of Pd nanostructures with respect to the irradiation dose. The error bar comes from the instrumental error of the profilometer. (c) *I*–*V* characteristics for Pd nanostructures fabricated at indicated doses. (d) Electrical resistivity of Pd nanostructures as a function of the ion dose.

In order to optimize the ion dose in relation to the electrical resistivity, current-versus-voltage (*I*–*V*) curves at room temperature were recorded. These measurements consist of applying a fixed current through the two outer electrodes while measuring the voltage drop across the two inner electrodes. For that purpose, Pd nanostructures patterned with the design illustrated in Figure 2a were fabricated from a spin-coated film of 110 nm in thickness. Two different ion beam currents were used to cover the full 2–100 μC/cm^2^ range: 7.7 pA for 2, 4, 6, 8, and 10 μC/cm^2^ and 24 pA for 20, 30, 40, 50, 60, 80, and 100 μC/cm^2^. The thickness of each nanostructure was measured by profilometry and is represented in Figure 2b. As represented in Figure 2c, a linear *I*–*V* dependence is observed for ion doses higher than 6 μC/cm^2^, that is, for the whole range studied except for the lowest doses, 2 and 4 μC/cm^2^ (the linear *I*–*V* curve corresponding to the 6 μC/cm^2^ ion dose is not included here for the sake of clarity, but it is shown in Figure S2). The linear fitting of the *I*–*V* curves indicates that the value of the electrical resistance decreases as the ion dose increases and then saturates at higher doses. Combining the electrical resistance together with the dimensions of the Pd nanostructures, the electrical resistivity was calculated and is represented in Figure 2d. This plot shows how the electrical resistivity decreases sharply as the ion dose increases until flattening out at a value of (70 ± 5) μΩ·cm for the dose of 30 μC/cm^2^.

### Compositional Analysis by STEM-EDS and the XPS Study

**Figure 3:**
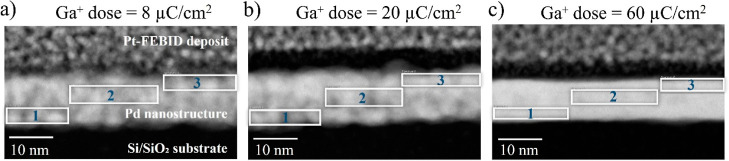
HAADF images of three selected samples that correspond to Pd nanostructures grown with Ga^+^ doses of (a) 8 μC/cm^2^, (b) 20 μC/cm^2^, and (c) 60 μC/cm^2^. During the lamellae preparation, the Pd nanostructure was protected with Pt by Focused Electron Beam Induced Deposition (FEBID). The dark layer on top of the Pd nanostructure corresponds to the interlayer formed between the Pd nanostructure and the Pt-FEBID deposit. The white rectangles indicate the area where the atomic composition was investigated by EDS.

In order to carry out structural characterization by TEM, cross-sectional lamellae of Pd nanostructures fabricated under Ga^+^ doses of 8, 20, and 60 μC/cm^2^ were extracted. HAADF images and EDS measurements were carried out by using the STEM mode. First, based on HAADF images (Figure 3) the as-fabricated nanostructures exhibit very low roughness and absence of voids at analyzed areas. Moreover, a comparison of these images indicates that higher doses result in brighter nanostructures, which is attributed here to the presence of more agglomerated metallic grains. Second, EDS experiments were performed in three different regions for each nanostructure (indicated with white rectangles in Figure 3) in order to investigate their atomic composition.

Atomic contents of Pd, C, and O are collected in Table 1. According to these data, the palladium content is slightly lower for the structure grown with an ion dose of 8 μC/cm^2^, and higher for those grown through doses of 20 and 60 μC/cm^2^. Importantly, Ga^+^ implantation is negligible due to the low ion doses used for the fabrication of these nanostructures.

**Table 1:** Elemental Quantification of Pd, C, and O Obtained From Regions Indicated in Figure 3

Ga^+^ dose [μC/cm^2^]	region	Pd [at. %]	C [at. %]	O [at. %]
8	1	45 ± 0.2	50 ± 4	5 ± 3
	2	46 ± 0.2	49 ± 4	5 ± 3
	3	46 ± 0.2	49 ± 4	6 ± 3
20	1	65 ± 0.2	28 ± 11	7 ± 3
	2	59 ± 0.2	36 ± 6	5 ± 3
	3	45 ± 0.2	50 ± 4	5 ± 3
60	1	53 ± 0.2	41 ± 5	6 ± 3
	2	52 ± 0.2	44 ± 4	4 ± 3
	3	48 ± 0.2	50 ± 4	2 ± 7

As recently reported, the irradiation of Pd_3_(OAc)_6_ thin films with an electron beam produces the decomposition of the organometallic precursor, varying the Pd-oxidation state and therefore the electrical resistivity associated with the resulting nanostructures by up to 2 orders of magnitude. In particular, the reduction of Pd^2+^ into metallic palladium, Pd^0^, occurs when applying a dose of 30,000 μC/cm^2^.^36^ In order to quantify this conversion for Ga^+^ irradiated films, the Pd^2+^ conversion into Pd^0^ in ion-irradiated films, an XPS study was performed. The obtained results are gathered in Figure S3. Briefly, when irradiating Pd_3_(OAc)_6_ thin films with a Ga^+^ beam by directly applying the smallest dose (8 μC/cm^2^), ∼95% of the starting Pd^2+^ is reduced to Pd^0^, and the same applies to the other doses. Combining the information obtained from the electrical measurements and from the compositional analysis, the fabrication of Pd nanostructures for the applications presented below was performed by applying doses in between the optimal range of 20–40 μC/cm^2^. The use of these low ion doses makes negligible potentially focused ion beam side effects such as thermal implications, implantation, or halo effects due to sputtering.

### BCA-Based Simulations to Model the Evolution of the Precursor Layer

**Figure 4:**
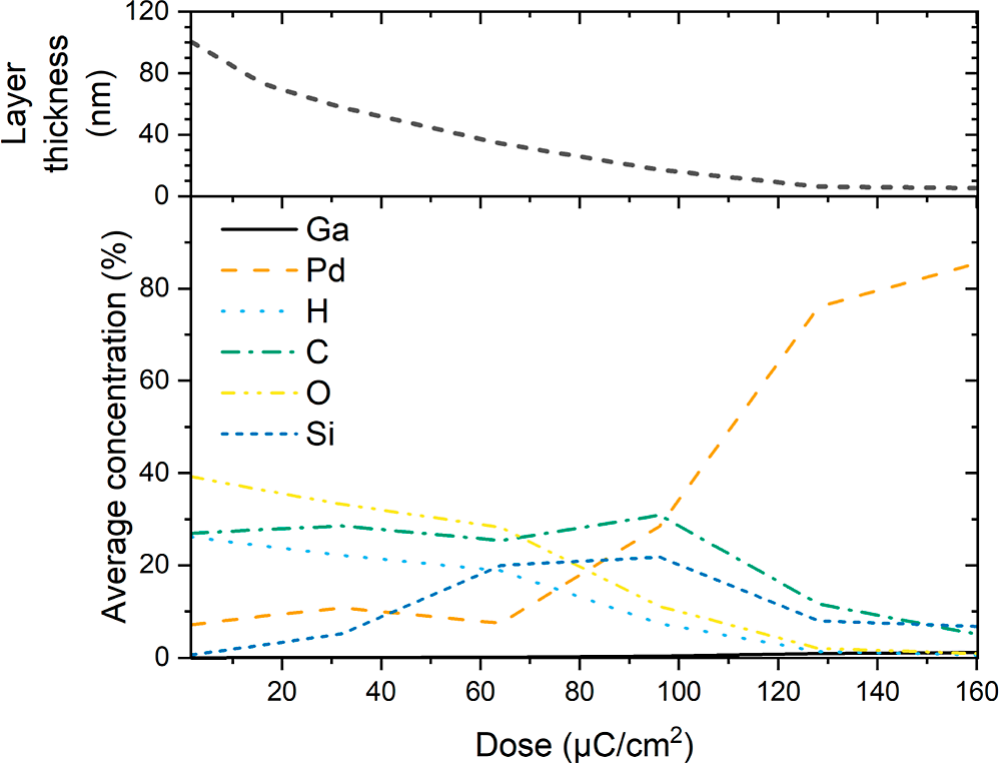
Evolution with Ga^+^ dose of the average Pd composition and the thickness of the precursor layer modeled with SDTrimSP.

Theoretical calculations were performed to provide further understanding of the processes occurring upon Ga^+^ irradiation of the Pd_3_(OAc)_6_ thin film. The SDTrimSP simulations show that the average Pd concentration (at. %) increases gradually with the Ga^+^ ion dose to reach a maximum value of 85% at the maximum dose of 160 μC/cm^2^ (Figure 4). For a dose of 100 μC/cm^2^, the Pd concentration is equal to 28.5% when considering all elements or equal to 40.4% when considering only Pd, C, and O (*i.e.,* the elements that have also been detected by EDS, see Table S1). In the latter case, a good agreement between the simulations and the experimental results is achieved. The desorption of the volatile compounds and the sputtering of all compounds lead to a decrease in layer thickness. With increasing dose, the sputtering becomes dominated by the partial sputtering yield of Pd (Figure S4). It is also important to note that the definition of the interface position, that is, for a Si concentration of 50%, leads to a maximum average concentration of about 20% in this layer. This can be explained by the mixing of other elements, especially H and O, into Si. More information on the composition of the layer and its evolution with irradiation dose can be seen in Figure S5. This value could be affected by the implementation of diffusion into the SdTrimSP code; that is, the diffusion of the light precursor elements into the silicon substrate could be overestimated. According to the layer thickness evolution, the thickness decreases when the Ga^+^ irradiation dose increases, which agrees with the experimental results observed during the characterization of our Pd nanostructures (Figure 2b).

**Figure 5:**
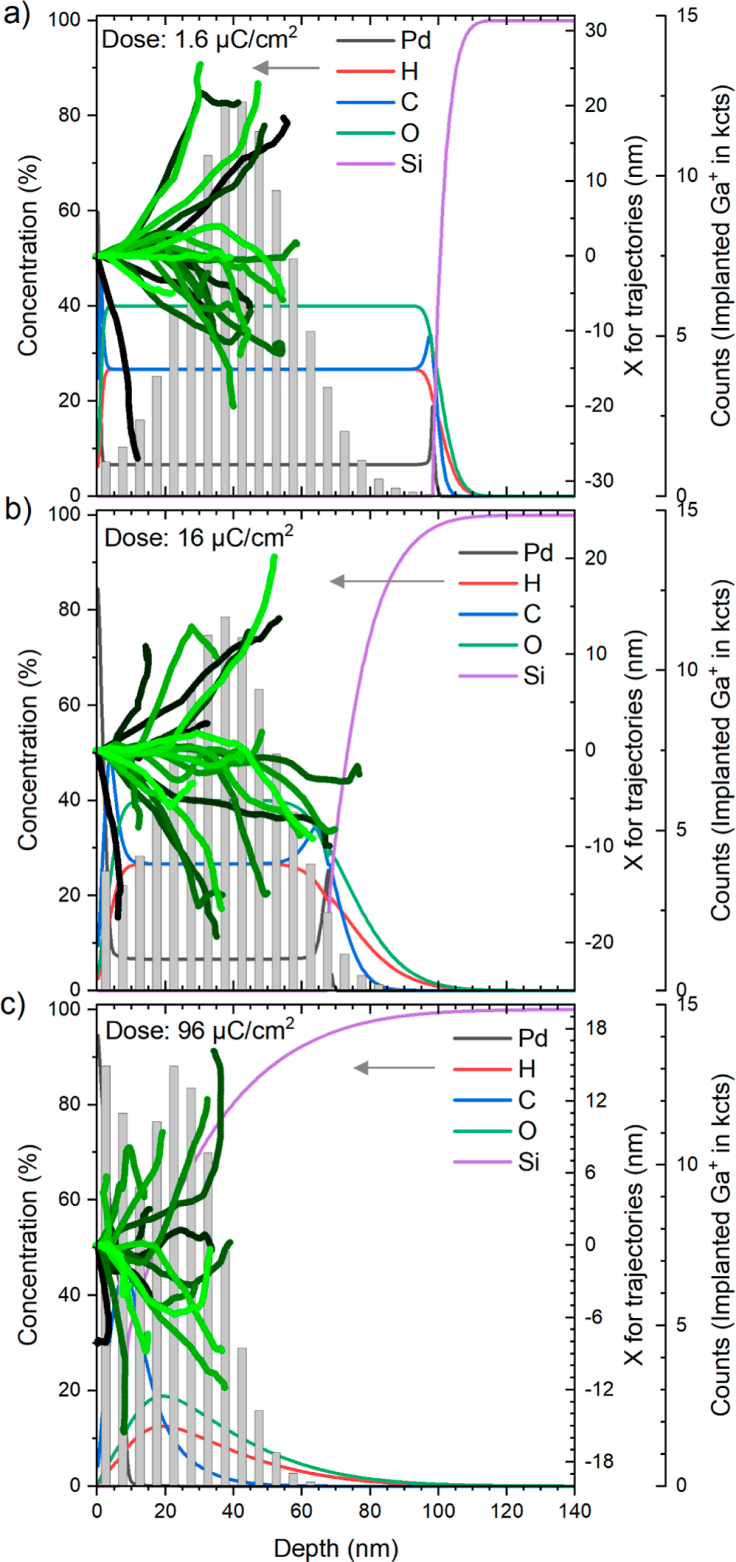
Different graphs obtained by SDTrimSP simulations to compare sample compositions as a function of depth (1st *y*-axis), trajectories of Ga^+^ ions in different shades of green (2nd *y*-axis), and the implantation depth of Ga^+^ as a histogram (3rd *y*-axis) for the Ga^+^ doses of (a) 1.6 μC/cm^2^, (b) 16 μC/cm^2^, and (c) 96 μC/cm^2^.

At the beginning of the Ga^+^ irradiation, the composition of the precursor layer is homogeneous with respect to depth. However, the implantation depth of the 30 kV ion beam is not high enough to process the whole layer right from the beginning (Figure 5a), leading to some accumulation of Pd at the sample surface and some modification of the precursor composition at the precursor/Si interface, that is, some accumulation of Pd and C at the interface (Figure 5b). Theoretically, only roughly from a dose of 16 μC/cm^2^, the energy of the Ga^+^ ions is deposited into the whole precursor layer. At the highest dose used in the simulation (Figure 5c), a significant amount of the Ga^+^ ion energy is deposited into the Si substrate. Due to the difference in density, the implantation depth of the Ga^+^ ions decreases with an increasing dose. Detailed information on the sample composition for the different fluences can be found in Figure S5. The higher Ga^+^ doses required to reach Pd concentrations in the 40% range in the simulations compared to the experimental values can be explained by underestimating the ejection of volatile components in SDTrimSP. This has been taken into account to some degree by applying certain diffusion coefficients to H, C, and O. Applying higher diffusion coefficients would not have solved the problem but would have produced even higher Pd concentrations at larger doses (see Table S1), which is contrary to the experimental results where Pd concentrations stay stable over a given dose range (Table 1). The inability of SDTrimSP to reproduce this behavior correctly can be attributed to the binary collision approximation, which takes into account only the interaction between a moving ion or recoil atom and a single target atom without including many-body effects that allow for the description of more complex chemical environments.

As a proof of concept, Pd microstructures were fabricated as top electrical contacts on a Pt nanowire. To achieve this objective, first, a Pt nanowire was directly grown by the FIBID (focused ion beam-induced deposition) technique on a Si/SiO_2_ substrate, correctly positioned with respect to pre-patterned alignment marks. Second, after the spin-coating process, the organometallic film was irradiated following four patterns to be used as electrical contacts onto the nanowire. Finally, non-exposed film areas were removed by immersing the sample in CHCl_3_, and the nanowire was measured by the four-probe technique at room temperature. The Pt–C nanowire remained after the spin-coating process, film irradiation, and developing step without appreciating any change in either the morphology or the electrical properties.

**Figure 6:**
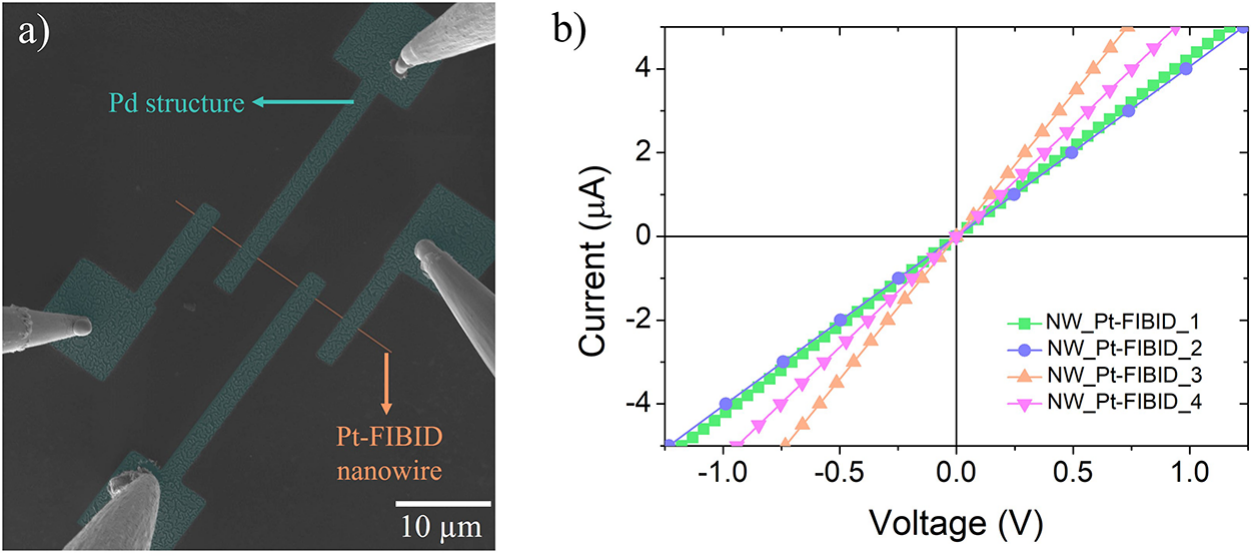
(a) Artificially-colored SEM micrograph of one of the measured Pt nanowires with the four electrical Pd contacts. This device corresponds to a sample named as “NW_Pt-FIBID_3”. (b) *I*–*V* plot, indicating resistances in the range of 147–247 kΩ.

Figure 6a shows one of the measured devices where the Pt-FIBID nanowire is artificially colored in orange (dimensions of 30 μm × 100 nm × 30 nm) and the Pd-based electrical contacts in blue. All Pt-FIBID nanowires were grown under the same conditions (30 kV of beam voltage and 1.1 pA of beam current) and electrically measured and are represented in Figure 6b. The four Pd-based electrical contacts were fabricated under optimized conditions by varying the thickness of the initial spin-coated film and the ion dose (see Table 2). The fabrication of the four electrical contacts, having a total area of 580 μm^2^, required only ∼7.5 s of ion irradiation for the dose of 10 μC/cm^2^ using 7.7 pA and ∼7.25 s for the dose of 30 μC/cm^2^ using 24 pA. The final thicknesses of the electrical contacts are in the range of 15 to 30 nm, depending on the initial spin-coated film thickness.

**Table 2:** Growth Parameters for the Four Pd Electrical Contacts Here Studied Together With the Resistance and Resistivity Values for Each Pt Nanowire

sample	spin-coated initial thickness [nm]^a^	Ga^+^ dose for Pd microstructures [μC/cm^2^]	resistance [kΩ]	resistivity [10^3^ μΩ·cm]^b^
NW_Pt-FIBID_1	350	30	235	9.2
NW_Pt-FIBID_2	200	30	247	9.2
NW_Pt-FIBID_3	125	30	147	5.4
NW_Pt-FIBID_4	125	10	189	7.0

a Value corresponding to the initial thickness of the spin-coated film before the Ga^+^ irradiation.

b Value calculated considering that all nanowires have a width of 100 nm and a thickness of 30 nm.

The four devices were electrically measured by the four-probe technique at room temperature. The *I*–*V* plots exhibit a linear behavior for all nanowires and only slight variations in terms of resistance when the growth parameters of the Pd microstructures are changed. Table 2 gathers the electrical resistance and resistivity data corresponding to each nanowire. The measured electrical resistivities for these semiconducting Pt-FIBID nanowires correspond to values in the 5400–9200 μΩ·cm range, which is in good agreement with other similar Pt–C nanowires grown by FIBID.^42^ The very low processing time, the low ion-induced damage, and the low electrical resistivity of these Pd structures make them very promising in the field of electronics for their applicability to contacting other nanomaterials or nanostructures, if chemically compatible (2D materials, thin films, organic monolayers, *etc*).

### Planar Nanogaps Using Pd Microstructures

**Figure 7:**
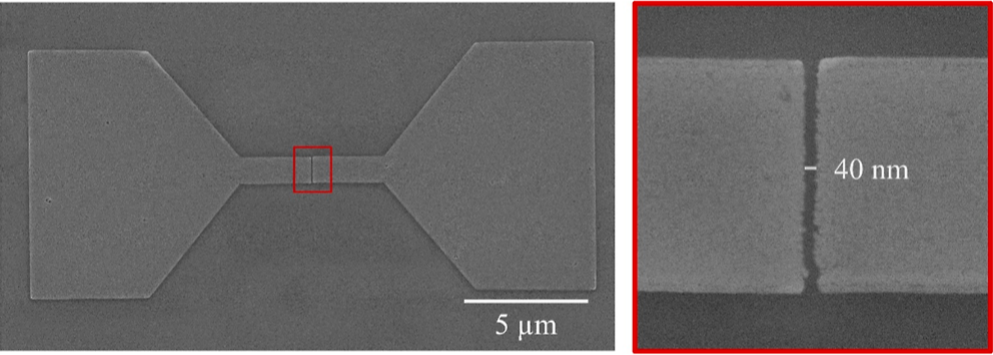
SEM micrograph of a planar nanogap device on a Si/SiO_2_ substrate with the two microstructures separated by 40 nm and grown in 26 s of ion irradiation.

The formation of planar nanogaps using Pd microstructures as electrodes has been undertaken. Two metallic Pd microstructures were horizontally placed facing each other and separated by only 40 nm. This gap value was chosen following the simulations on the ion trajectories shown in Figure 5b, which indicate ion lateral deviations within the ±20 nm range. The obtained resolution of 40 nm is comparable to other Ga-FIB-based processes,^43^ but it could be improved to sub-10 nm by using irradiation with focused He and Ne ions.^22^ In the latter case, the method would approach the best resolution achievable with more standard lithography techniques such as electron beam lithography.^20^ The structure shown in Figure 7, with a total area of 146 μm^2^, was grown in only 26 s by means of an Ga^+^ beam current of 1.1 pA, which corresponds to an ion dose of 20 μC/cm^2^. The initial spin-coating thickness was 215 nm, and the final thickness for this structure was 25 nm.

The high resolution achieved enables these structures to be used as nanogap electrodes for the fabrication of nanometer-size devices and circuits or to observe the electrical behavior of different nanomaterials.^44^ The main advantage of this fabrication method is the very low ion dose required to fabricate metal contacts and therefore the quick fabrication and negligible ion-induced damage in the sample. Thus, this approach is a very good option to fabricate metallic nanopatterns with nanogaps that could be applied to place top or edge contacts on 2D semiconducting materials, neither inducing damage nor leaving resist residues as it occurs with resist-based fabrication methods. Furthermore, electrical nanogap devices for molecular electronics and biosensing have gained importance in recent years. A growing interest in inserting single molecules between electrodes exists due to the importance of understanding the transport behavior at the level of a single molecule to prepare entirely molecular integrated circuits.^45,46^ For biosensing, the biomolecules are trapped inside the gap between the electrodes and are detected by measuring their electrical behavior. Considering the range of our gaps (∼40 nm), these devices could, for example, be bridged to gold nanoparticles, which are used as labels for ultrasensitive electronic detection.^47^

### Large-Area Mesh Fabrication and Electrical Characterization

**Figure 8:**
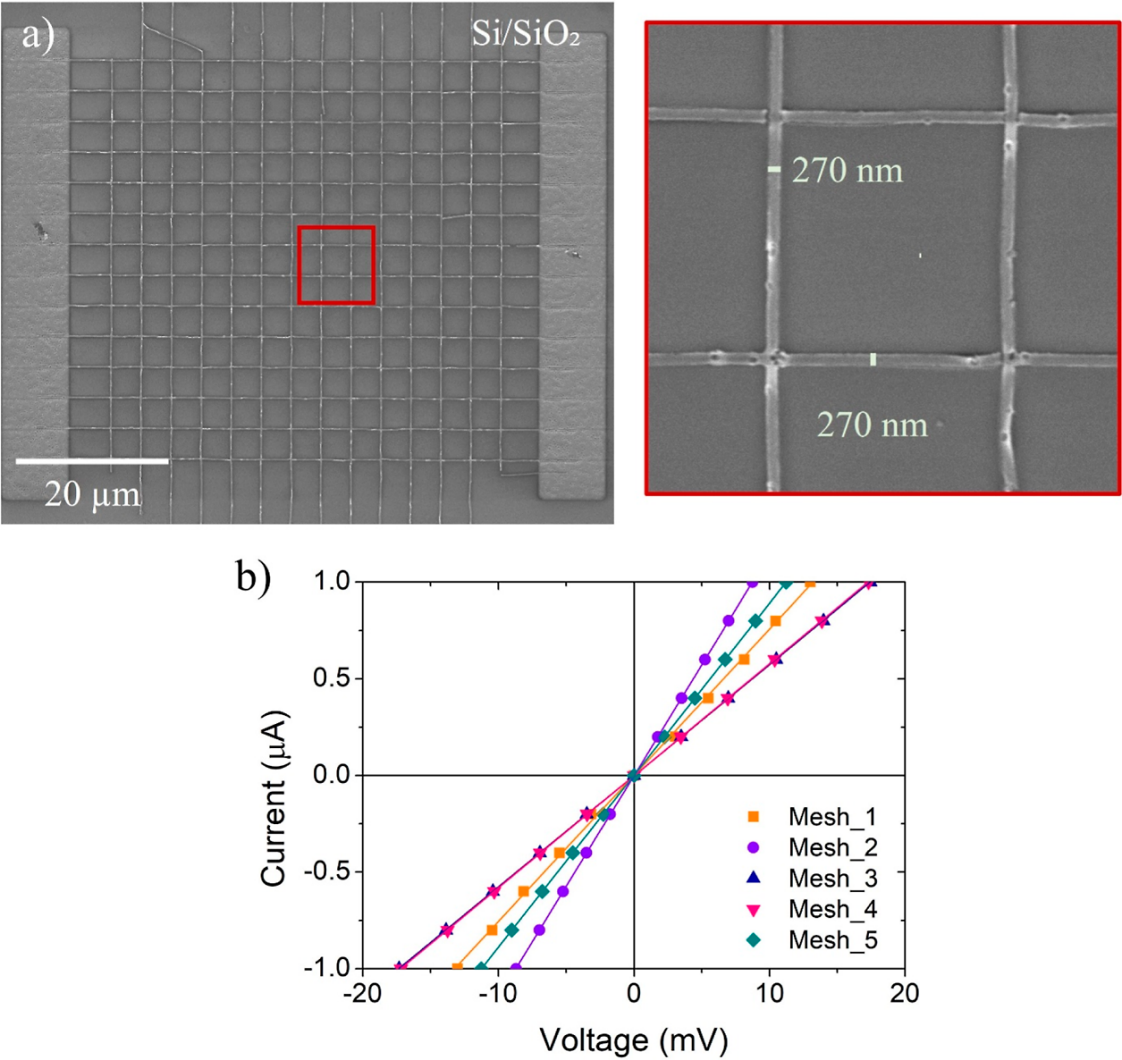
(a) SEM micrograph of a device formed by a large-area mesh and two electrical contacts fabricated in one step on a Si/SiO_2_ substrate. (b) *I*–*V* measurements of five devices indicating electrical resistances in the 8–17 kΩ range.

The third application of this research consists in the growth of large-area metallic meshes, where the scale and shape of the patterns are changed without modification of the metallic nature of the Pd structures. To that end, square-based grids were fabricated on Si/SiO_2_ substrates covered by ∼270 nm-thick spin-coated films, bearing two wider pads at both sides of the grid in order to facilitate electrical measurements through the electrical microprobes, as shown in Figure 8a. Each mesh was composed of a total of 28 patterns with dimensions of 90 μm × 200 nm. The total irradiation time is 2 min, using an ion beam current of 1.1 pA, which corresponds to an ion dose of 30 μC/cm^2^. The lateral electrical contacts, with dimensions of 75 μm × 10 μm, were fabricated using an ion beam current of 24 pA in only 30 s, which corresponds to an ion dose of 48 μC/cm^2^. Considering the irradiation times, each electrical device (mesh and two electrical contacts) took only 2.5 min, resulting in a final thickness of (25 ± 5 nm). Figure 8b shows the *I*–*V* curves registered by the two-probe technique at room temperature. A linear Ohmic behavior is noticed in all devices fabricated under the same conditions. The electrical resistance values for these devices were in the 8–17 kΩ range.

With a view to expanding the scope of these metallic micro- and nano-structures toward their implementation in research areas in which optical transparency is required, we tested the fabrication of large-area meshes on indium tin oxide (ITO) substrates, obtaining promising results as shown in Figure S6.

## Outlook and Conclusions

Micro- and nano-lithography techniques for large-area patterning commonly rely on sacrificial spin-coated resists that are removed at an intermediate step. There are very few examples where the spin-coated film is functional and is not removed during the lithography process. One of these rare examples is the case of 3D polymer-based scaffold structures for cell growth proliferation^48^ or polymers structured to serve as mechanical cantilever resonators,^49^ both fabricated by two-photon lithography. Another example is that of 3D cryogenic electron-beam writing for optical applications.^50^ The advantage of an electrically functional spin-coated film like Pd_3_(OAc)_6_ is the simplicity of the overall process, consisting in only three steps: spin coating the substrate with a Pd precursor film, focused Ga^+^ irradiation, and film development, as illustrated in Figure 1. This is a significant improvement with respect to previous strategies using organometallic films, where post-annealing steps were always needed to reach a metallic behavior. Remarkably, the patterning resolution achievable with the focused Ga^+^ beam irradiation is very high: gaps of 40 nm have been shown here, but the potential for better resolution through further optimization of the working parameters or by means of lighter focused ions (He, Ne, *etc.*) is very high.^51^ Moreover, the fact that the required ion dose to create metallic patterns (with electrical resistivity values of 70 μΩ·cm) is very low, which is 30 μC/cm^2^, makes this approach very efficient with respect to other direct-write lithography techniques. As a comparison, the growth of metallic structures by focused electron and ion beam-induced deposition requires charge doses several orders of magnitude higher.^38^ This translates into a much shorter processing time for growing these Pd structures. For example, the Pd microstructure shown in Figure 7, with a total area of 146 μm^2^, only took 26 s of irradiation compared to several hours of irradiation if grown by focused electron/ion beam-induced deposition. Cryogenic-focused ion beam-induced deposition is another high-throughput direct-write lithography technique that uses a similar ion dose range as the one used for these films, but it requires lowering the temperature of the stage to cryogenic temperatures.^52^

The results shown here are the first of their kind and are thought to have important implications. First, they pave the way for their straightforward use in various applications where metallic micro- and nano-patterns are needed, such as interconnects, conductive micro- and nano-meshes, metallic micro- and nano-templates, nanogaps, electrical contacts to nano-objects, and so forth. Second, these Pd_3_(OAc)_6_ films can also be decomposed by electron irradiation^34,36^ or light irradiation,^53^ offering the capability of applying on the same film several micro- and nano-lithography techniques that span a wide range of lateral sizes. Third, there exist other acetates and organometallic films that contain elements such as Au, Co, Fe, and so forth, and their patterning by focused ion beam irradiation could lead to structures exhibiting additional functional properties beyond electrical conductivity, such as plasmonic or magnetic behavior. Moreover, Al- and Cu-based resists with comparable behavior to Pd_3_(OAc)_6_ could be relevant for the growth of CMOS-compatible metallic structures.^54^

In summary, an efficient direct-write lithography method based on focused Ga^+^ irradiation to create Pd-rich metallic patterns out of spin-coated Pd_3_(OAc)_6_ films which does not involve any post-annealing step has been described here. The method exhibits high resolution, as demonstrated by the creation of structures with 40 nm gaps. The patterned structures, which present a low electrical resistivity value of (70 ± 5) μΩ·cm, have been used to contact a nanowire and measure its electrical properties as well as for creating proof-of-concept large-area metallic meshes. Theoretical simulations have shed light on the ion-induced processes leading to the growth of Pd-rich nanostructures.

## Supporting Information Available

Additional characterization details, AFM characterization, *I*–*V* curves, thickness measurements, XPS studies, other SEM micrographs, and additional information about Monte Carlo simulations (PDF)
