# Supplementary material for: High-Throughput Direct Writing of Metallic Micro- and Nano-Structures by Focused Ga+ Beam Irradiation of Palladium Acetate Films
Source: ACS Appl Mater Interfaces. 2022 Jun 7;14(24):28211–20. doi: 10.1021/acsami.2c05218 (PMC9227716; doi:10.1021/acsami.2c05218)
Supplement: Supplementary file 1 — am2c05218_si_001.pdf [file am2c05218_si_001.pdf]

Supporting Information

High-throughput direct writing of metallic micro- and nano-structures by focused Ga^+ beam irradiation of palladium acetate films

Alba Salvador-Porroche [a], Lucía Herrer [a], Soraya Sangiao [a,b], Patrick Philipp [c], Pilar Cea [a,b] and José María de Teresa [a,b].*

[a] Instituto de Nanociencia y Materiales de Aragón (INMA), CSIC-Universidad de Zaragoza, 50009 Zaragoza, Spain.

[b] Laboratorio de Microscopías Avanzadas (LMA), Universidad de Zaragoza, 50018 Zaragoza, Spain.

[c] Advanced Instrumentation for Nano-Analytics (AINA), MRT Department, Luxembourg Institute of Science and Technology (LIST), 41 rue du Brill, 4422 Belvaux, Luxembourg

E-mail: deteresa@unizar.es

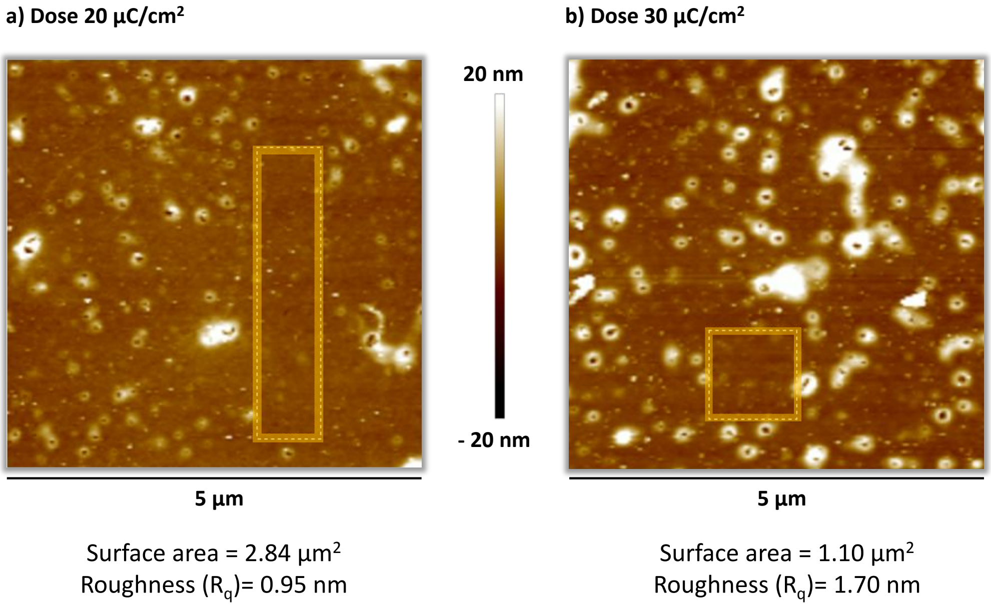

Figure S1: Estimation of the roughness value, pinhole-free areas have been analyzed by Atomic Force Microscopy, showing a root-mean-square roughness value in the range from 0.95 to 1.70 nm.

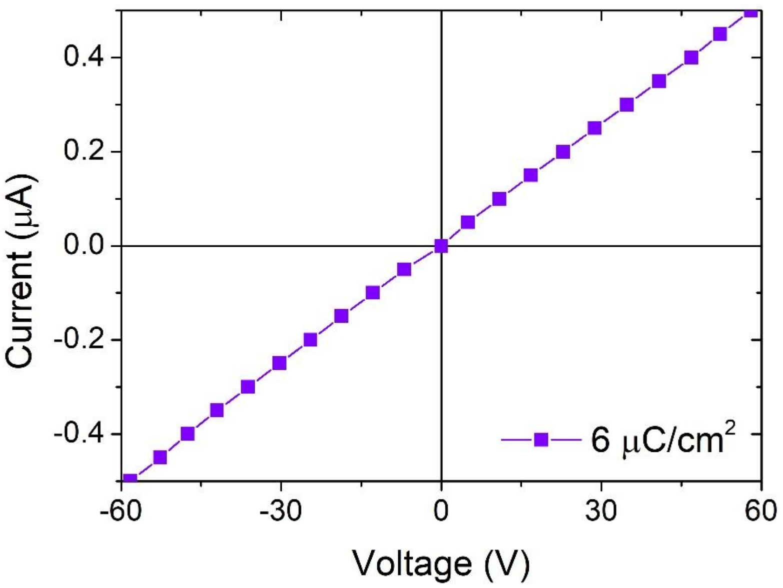

Figure S2. *I- V* curve registered for a Pd deposit fabricated with a dose of 6 μC/cm^2.

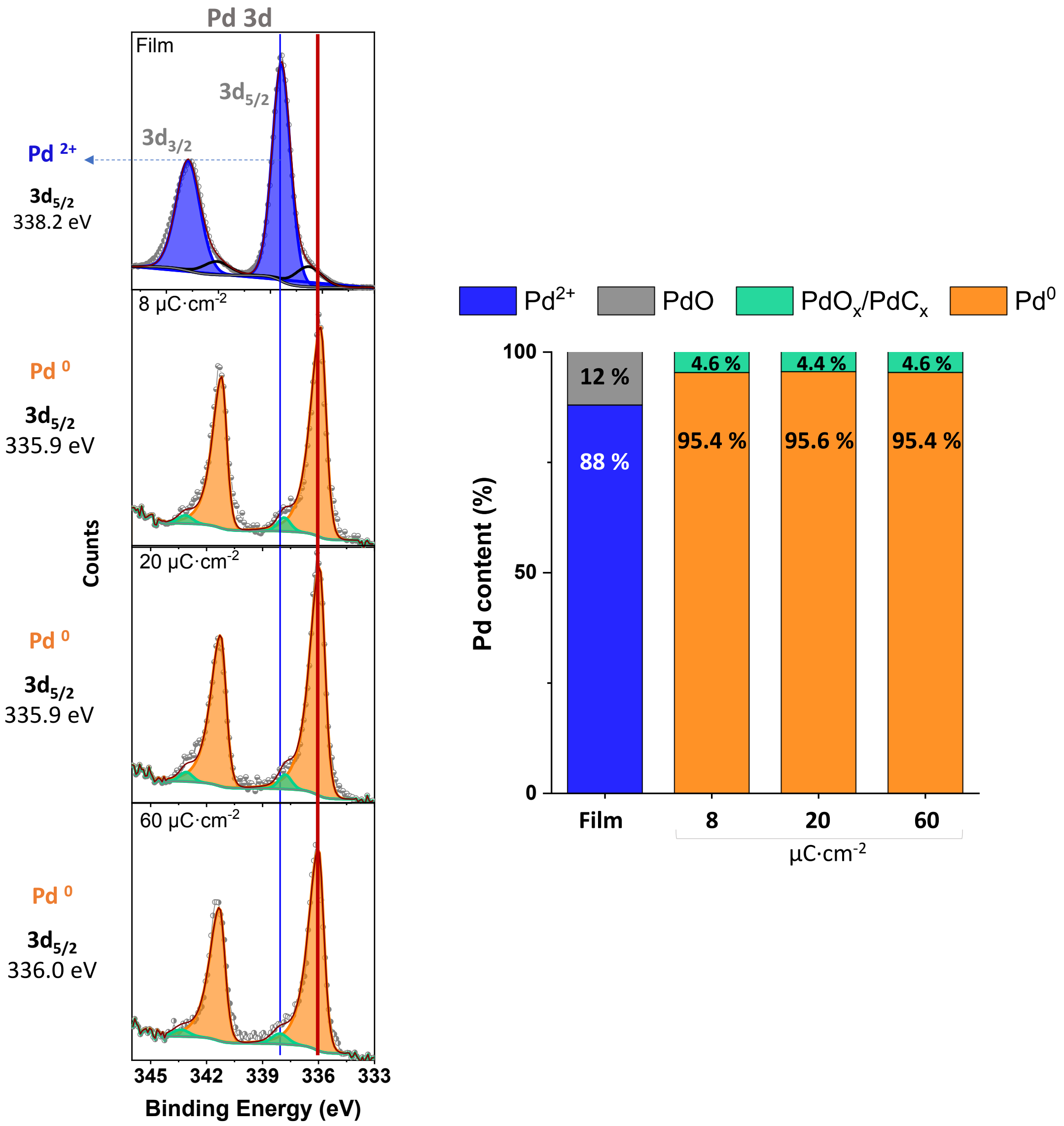

Figure S3. Left: Pd 3d XPS spectra for indicated samples and peak deconvolution. Right: % Pd content based on the % area corresponding the Pd $3d_{5/2}$ peak for each chemical Pd oxidation state at each sample.

Table S1: Average concentrations for the different doses when considering only Pd, C and O, i.e. the elements detected in EDS, according to the SDTrimSP simulations.

Ga^+ Dose [μC/cm^2]	Average concentration [at. %]		
	**Pd**	**C**	**O**
1.6	9.72	36.74	53.53
16.03	12.33	37.98	49.69
32.50	14.88	39.31	45.82
64.1	12.19	41.60	46.21
96.15	40.43	43.81	15.76
128.21	84.81	12.95	2.24
160.26	93.50	5.48	1.02

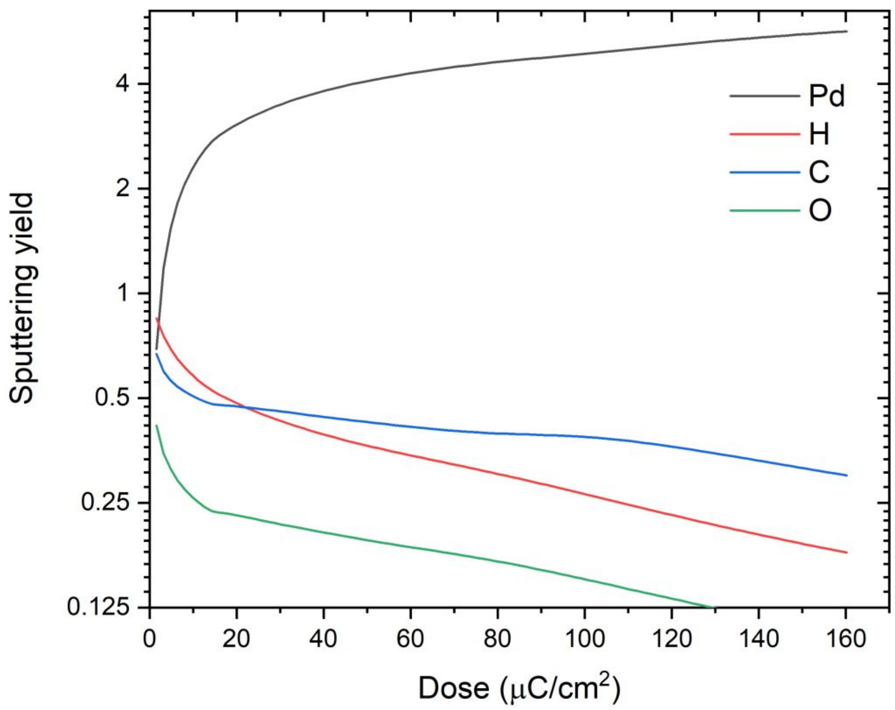

Figure S4: Sputtering yields for the chemical elements of the precursor molecule as a function of Ga dose. The removal of the light elements, i.e. H, C and O leads to an increase of the Pd sputtering yield. Diffusion followed by desorption leads in addition to sputtering to the removal of H, C and O from the precursor layer.

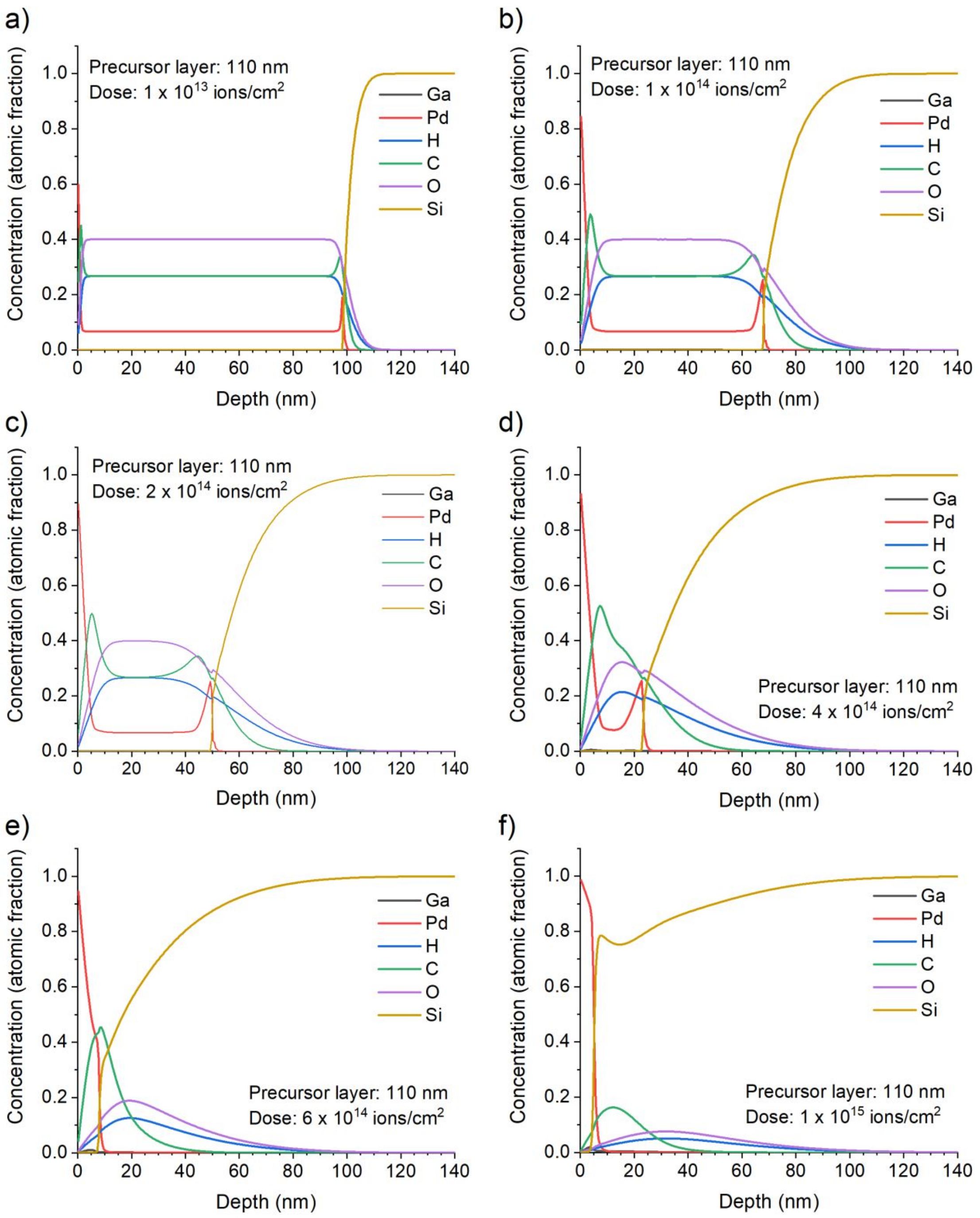

Figure S5. Depth profiles showing the sample composition at a specific dose obtained by SDTrimSP for Ga^+ irradiation of doses of a) 1.6 μC/cm^2, b) 16 μC/cm^2, c) 32 μC/cm^2, d) 64.1 μC/cm^2, e) 96.1 μC/cm^2, and f) 160.3 μC/cm^2. Starting from a dose of 32 μC/cm^2 the composition of the precursor layer is no longer homogeneous.

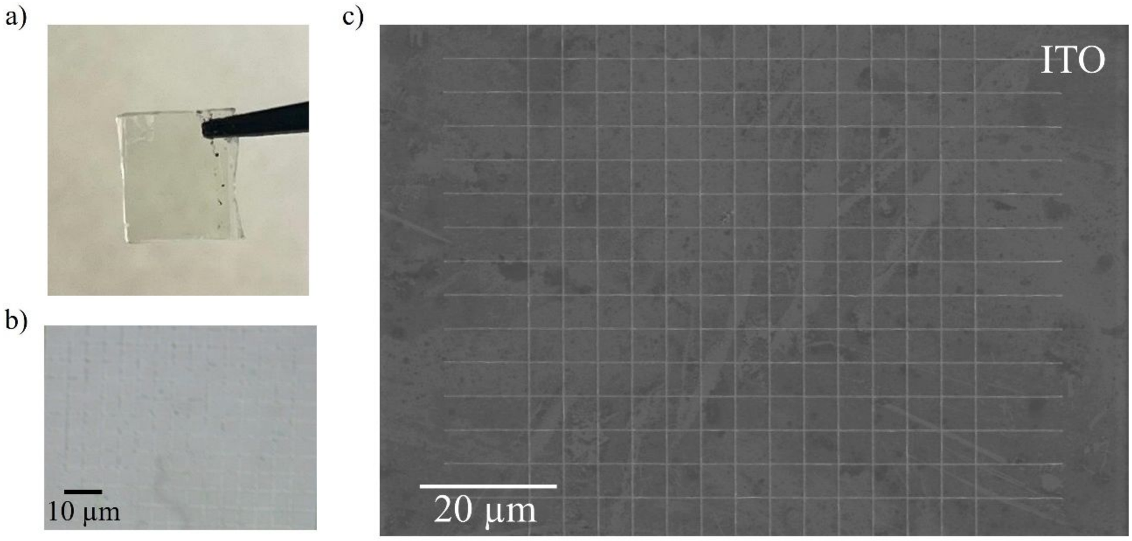

Figure S6. Fabrication of a large-area mesh on a transparent substrate (ITO). a) View of the substrate after the fabrication process. Micrographs of the fabricated mesh by b) optical microscopy, and c) SEM image of the modified ITO substrate.
